# Optimization with Response Surface Methodology of Microwave-Assisted Conversion of Xylose to Furfural

**DOI:** 10.3390/molecules25163574

**Published:** 2020-08-06

**Authors:** Carmen Padilla-Rascón, Juan Miguel Romero-García, Encarnación Ruiz, Eulogio Castro

**Affiliations:** 1 Department of Chemical, Environmental and Materials Engineering, Universidad de Jaén, Campus Las Lagunillas, 23071 Jaén, Spain; cpadilla@ujaen.es (C.P.-R.); jrgarcia@ujaen.es (J.M.R.-G.); ecastro@ujaen.es (E.C.); 2 Centre for Advanced Studies in Earth Sciences, Energy and Environment (CEACTEMA), Universidad de Jaén, Campus Las Lagunillas, 23071 Jaén, Spain

**Keywords:** lignocellulosic material, xylose, furfural, iron chloride, microwave reactor, biorefinery

## Abstract

The production of furfural from renewable sources, such as lignocellulosic biomass, has gained great interest within the concept of biorefineries. In lignocellulosic materials, xylose is the most abundant pentose, which forms the hemicellulosic part. One of the key steps in the production of furfural from biomass is the dehydration reaction of the pentoses. The objective of this work was to assess the conditions under which the concentration of furfural is maximized from a synthetic, monophasic, and homogeneous xylose medium. The experiments were carried out in a microwave reactor. FeCl_3_ in different proportions and sulfuric acid were used as catalysts. A two-level, three-factor experimental design was developed for this purpose. The results were further analyzed through a second experimental design and optimization was performed by response surface methodology. The best operational conditions for the highest furfural yield (57%) turned out to be 210 °C, 0.5 min, and 0.05 M FeCl_3_.

## 1. Introduction

Owing to the depletion of fossil resources, it is necessary to look for renewable sources for the production of fuels and chemicals. Lignocellulosic biomass is an alternative natural resource that presents multiple advantages, because of its abundance, renewability, low cost, and the fact that it is a waste product with no competing uses. Lignocellulosic materials are mainly composed of hemicellulose, cellulose, and lignin. Other fractions also present in a lower proportion are extractives, which are low molecular weight organic compounds, and the ashes, which are inorganic compounds. Although the chemical composition of the biomass is variable, hemicellulose is usually the second most abundant compound with multiple uses, after cellulose. Xylose is the most abundant pentose in the hemicelluloses of hardwoods and agricultural plants [1].

In recent years, the production of furfural from renewable sources has gained great attention within the concept of sustainable biorefineries, giving added value to lignocellulosic materials and advancing the sustainable bioeconomy. The U.S. Department of Energy identified furfural as one of the top 30 platform chemicals [2]. Its composition, formed by unsaturated bonds and aldehyde groups, makes it a versatile platform molecule, from which a great variety of chemical compounds can be obtained, with applications in oil refining, production of plastics, food, and pharmaceutical and agricultural industries [3,4,5].

Currently, the most commonly used lignocellulosic biomasses for production of furfural are corn cob, sugar cane bagasse, rice hull, and wheat bran, considering that they are rich in hemicelluloses and are available in large quantities [1]. Olive subproducts, specifically olive stones or pits, have also been used to obtain furfural [6]. Among the advantages of using olive pits, the high availability in Mediterranean areas, their composition with a high proportion of hemicelluloses, and their easy acid hydrolysis are frequently cited.

**Figure 1:**
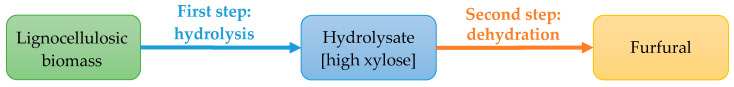
Schematic diagram of furfural production from lignocellulosic biomass.

Furfural is obtained from lignocellulosic materials in two steps, as shown briefly in Figure 1. The first step reaction, hydrolysis, consists of the transformation of polysaccharides into monosaccharides, obtaining a hydrolysate rich in sugars; in this case, the predominant sugar is xylose. The second step is the dehydration reaction of pentoses, mainly xylose, to furfural. This process can be done within the same reactor, one-stage, or separately in two steps. When the process is carried out in two steps, the hydrolysate can be separated from the insolubilized solid after the first step, allowing greater utilization of the lignocellulosic material. Furfural is obtained by dehydration of the pentoses (mainly xylose) and the unhydrolyzed solid can be employed for other uses, such as enzymatic hydrolysis and fermentation [7].

The pentose dehydration reaction involves the protonation of three carbon atoms in the sugar ring, removing three water molecules and obtaining the furanic molecules. Normally, these reactions take place in an aqueous medium, owing to its polarity, availability, sustainability, and low price. In the dehydration reaction, undesired secondary reactions also occur, by condensation between the furfural and the intermediate products of dehydration and by the degradation of the pentoses to low molecular weight products, generating soluble and insoluble secondary products (called humins) [8]. The use of catalysts facilitates the dewatering reaction. Most of the catalysts studied have Brønsted sites, which directly dehydrate the initial carbohydrate. Furthermore, the use of Lewis acid sites favors the isomerization of the carbohydrate and the subsequent dehydration through the Brønsted sites. These catalysts can be homogeneous, the disadvantages of which are high pressure and the production of a corrosive acid stream that needs to be neutralized. Alternatively, heterogeneous catalysts, limited by their cost and synthesis, are also used and may be subjected to deactivation owing to the deposit of subproducts on their surface [9,10].

Hemicellulose-rich raw materials can be hydrolyzed by various processes, among which the dilute acid hydrolysis stands out as one of the most efficient to selectively recover hemicellulose sugars [7,11,12]. These acids, like sulfuric acid, also enhance the dehydration reaction, in which it acts as Lewis acid [13]. Furthermore, the potential of salts as a catalyst for these reactions has been corroborated in multiple studies. Metal salts, and especially metal chlorides, such as FeCl_3_, have been used in the pretreatment step of lignocellulosic materials to improve the generation of sugars that can in turn be converted into valuable chemicals, acting also as a catalyst for the dehydration of sugars into furfural, as a prominent example. According to the proposed mechanism by which this general process takes place, FeCl_3_ acts as Brønsted acid in several reactions whose equilibrium is influenced by the pH and the initial concentration of the metal salt, improving the performance in obtaining furfural [4,14,15]. A complete description of the way metal salts can act for pretreating lignocellulosic materials along with relevant examples is available elsewhere [16].

Microwave-assisted reactions are booming, as they have many advantages, for example, they are versatile, need shorter reaction times, present uniform heating, and can be more efficient reactions. In multiple studies, microwave-assisted heating resulted in better yields compared with traditional heating methods [10,17,18]. Microwaves are widely used with lignocellulosic materials for the production of value-added chemicals such as furfural [5,19]. Another application is the fractionation of lignocellulosic materials. For example, Zhang et al. [20] reported on a microwave-assisted organoslv pretreatment using raw poplar. In another study, rye and wheat stillage were submitted to a first microwave treatment to produce a highly concentrated sugar solution, which was further fermented to ethanol [21]. Microwaves were also used to assist hemicellulose extraction from corn fiber [22], or for extracting phenolics from cocoa pod husk [23]. Recently, microwave-assisted treatment by deep eutectic solvents was reported for the delignification of garlic skin [24].

Response surface methodology is a tool that allows obtaining a mathematical model to establish a relationship between the parameters of interest studied (temperature, time, and so on) and the results obtained (yields, conversions, and so on). The interrelationships between the factors studied and their influence on the responses obtained are analyzed statistically to determine their significance. It also allows finding the best experimental conditions based on the results. The use of this methodology is widespread when optimizing the process conditions for furfural production [25,26,27].

As a previous step to get useful information on the best conditions to produce furfural from the pentose fraction of lignocellulosic materials, this paper addresses the use of sulfuric acid and ferric chloride to obtain furfural from a solution with a high concentration of xylose (30 g/L) in a monophasic and homogeneous medium. The microwave heating technique is used because of its advantages. The main objective is to optimize the conditions for the highest furfural production. For this purpose, two experimental designs were made, with temperature, time, and concentration of ferric chloride as factors.

## 2. Results

### 2.1. Two-Level Factorial Design

The results obtained from the factorial design of 11 experiments (2^3^ + 3 central points) with the three factors studied (concentration of FeCl_3_, time, and temperature) are shown in Table 1. The initial concentration of xylose was 30 g/L, while the consumption values ranged between 7.52 and 29.44 g/L, which is equivalent to conversion between 25.06 and 98.13% (above 90% in most cases). As for the furfural obtained, the values were between 3.02 and 10.35 g/L, which represents a yield of 15.63 and 53.59%, respectively. Finally, the selectivity ranged between 25.83 and 62.38%, with the average above 45%.

An overview of the results shows that increasing temperature also produced an increase in the xylose consumed and the furfural produced, indicating that the temperature favours the dehydration of xylose and the production of furfural, but it can also be seen that this does not only depend on the temperature factor. For determining the effect of the factors studied (concentration of FeCl_3_, time, and temperature) on yield (furfural yield), conversion (xylose conversion), and selectivity (furfural selectivity), the results were statistically analyzed and modelled according to a linear model with interaction between the factors. The models obtained in coded terms together with the *p*-value, *R*^2^, coefficient of variation (CV), and standard deviation (SD) are shown in Table 2. As can be seen, for all models, the *R*^2^ obtained is in the order of 0.99 (99% of the change is produced by the factors studied) and CV in all cases is lower than 3.2%.

**Figure 2:**
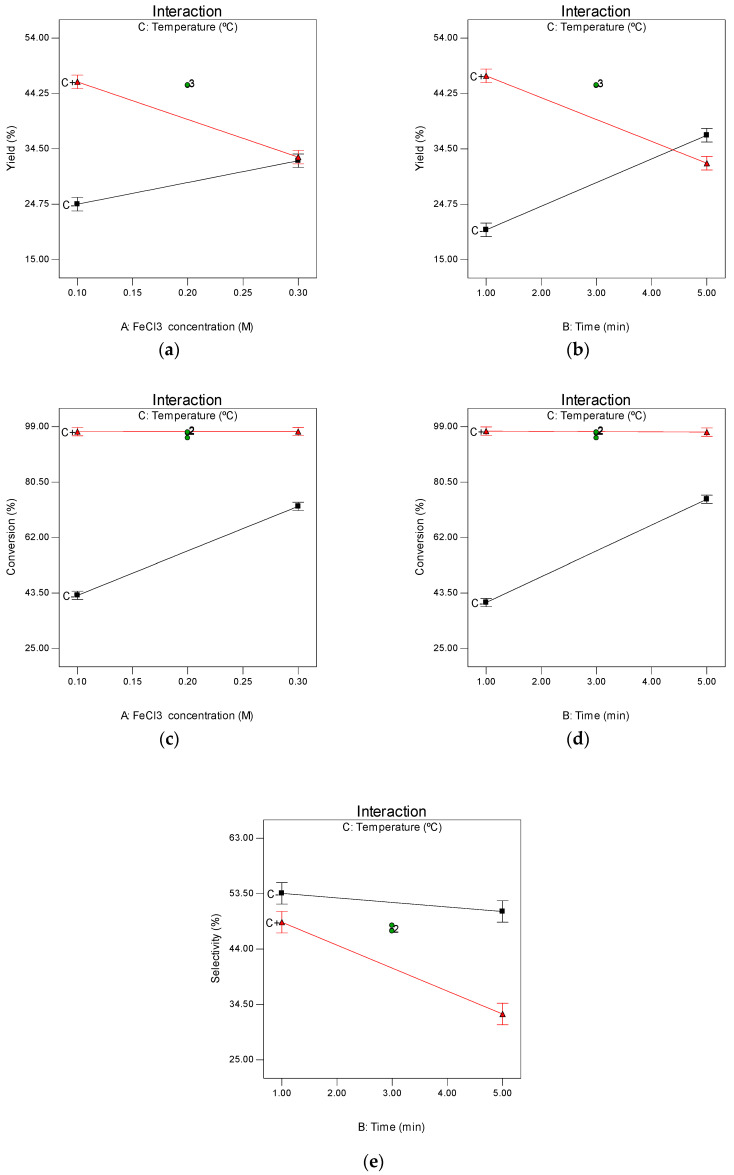
Interaction of factors studied in the factorial design: (**a**) interaction of FeCl_3_ concentration and temperature in yield; (**b**) interaction of time and temperature in yield; (**c**) interaction of FeCl_3_ concentration and temperature in conversion; (**d**) interaction of time and temperature in conversion; (**e**) interaction of time and temperature in selectivity. Red line, 200 °C; black line, 170 °C.

The temperature (C) is the most influential factor on the yield, as can be deduced from the highest value of its coefficient (contribution >20%); moreover, the total contribution of the temperature, if the interaction concentration FeCl_3_-temperature (AC) and the interaction time-temperature (BC) are taken into consideration, represents more than 75%. At a low temperature, the yield increases with the concentration of FeCl_3_ (A), while at a high temperature, the yield decreases with the increasing concentration of FeCl_3_. Similar yield results are obtained at high concentrations of FeCl_3_, independently of the temperature (Figure 2a). As far as the interaction between temperature and time is concerned, yields increase with time (B) in the low temperature region, while the behavior is the opposite at a high temperature (Figure 2b).

Regarding the conversion, a similar pattern to that in the yield was observed, that is, the temperature is the most influential factor. In this case, the contribution of the linear term is more than 52% and, together with AC and BC interactions, it represents 70%. In the case of conversion, the other two factors have a greater influence, contributing 17%. At a high temperature, the conversion is high regardless of the FeCl_3_ concentration and, at a low temperature, the conversion increases with the FeCl_3_ concentration, not reaching as high as at a high temperature (Figure 2c). The interaction between temperature and time follows the same trend described above for conversion (Figure 2d).

Finally, in the case of selectivity, the influence of the factors studied is different from the two previous cases, because the most influential factor is the FeCl_3_ concentration, with a contribution of more than 43%, with that of temperature and time being 27% and 19%, respectively. These last two factors interact in such a way that, at a low temperature, the selectivity decreases slightly over time and, at a high temperature, the selectivity decreases more drastically over time (Figure 2e).

When looking for the conditions that could maximize the variables studied only in the case of selectivity, we can say, without the need to make calculations, that they would be the lowest of all the factors studied ([FeCl_3_] = 0.1 M, time = 1 min, and temperature = 170 °C), because all the coefficients of the model obtained are negative (Table 2). In the case of yield and conversion, calculations would be necessary because, among the coefficients of their models, there are positive and negative values, but, if Figure 2 is observed, they can also be deduced. For the conversion, it can be easily deduced from Figure 2c,d that the best conditions are the high temperature (200 °C) and, in that case, the concentration of FeCl_3_ and time has little influence, so one would opt for low values ([FeCl_3_] = 0.1 M, time = 1 min). In the case of the yield, observing Figure 2a,b, it is also easy to deduce that the best conditions would be 200 °C, [FeCl_3_] = 0.1 M, and 1 min.

### 2.2. Central Composite Design

On the basis of the results obtained in the two-level factorial design, a rotatable composite central design was designed to further assess the influence of the selected factors and to optimize the conditions for obtaining furfural. The experimental range was changed to shorter times (0.5–1 min) and lower FeCl_3_ concentrations (0.05–1 M), and the temperature range was reduced to a shorter one (190–200 °C). The results (xylose consumption, furfural produced, yield, conversion, and selectivity) of the 25 experiments carried out according to the design conditions are shown in Table 3.

The initial concentration of xylose was maintained at around 30 g/L, resulting in this design displaying higher consumption values than those in the factorial design, ranging from 15.57 to 28.88 g/L or conversion of between 51.85 and 95.54%. As for the furfural obtained, values between 6.37 and 10.76 g/L were obtained, which represents a yield of 33.16 and 55.78%, respectively, a shorter range than in the factorial design and higher on average. Finally, the selectivity showed values between 55.84 and 66.23%, being on average around 61%, compared with the factorial design where it was around 46%.

For the analysis of the influence on the responses (yield, conversion and selectivity) of the factors studied (concentration of FeCl_3_, time, and temperature), the results were evaluated using the response surface methodology and modelled according to a quadratic model. The models obtained in coded and real terms together with the *p*-value, *R*^2^, coefficient of variation (CV), and standard deviation (SD) are shown in Table 4. The three models show *R*^2^ surpassing 0.91, yield and conversion above 0.97 (97% of the change is produced by the factors studied), and a CV in all cases below 2.5% (below 1.3 in the case of selectivity), that is, good values that allow us to use the models obtained for analysis.

**Figure 3:**
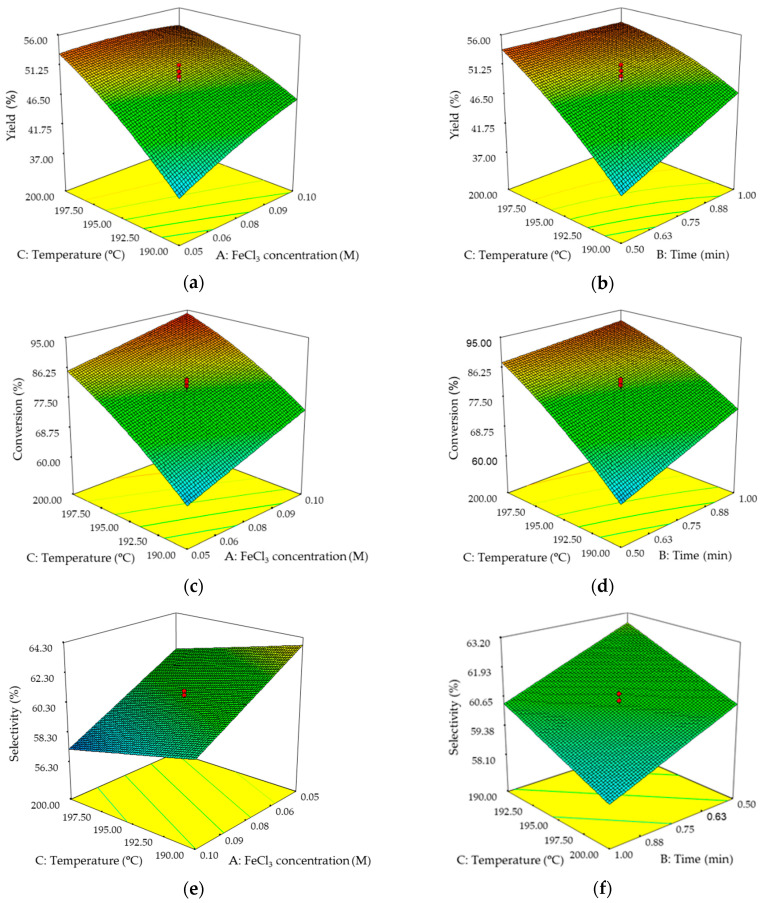
Response surfaces obtained for the composite central design: (**a**) influence of FeCl_3_ concentration and temperature on yield; (**b**) influence of time and temperature on yield; (**c**) influence of FeCl_3_ concentration and temperature on conversion; (**d**) influence of time and temperature on conversion; (**e**) influence of FeCl_3_ concentration and temperature on selectivity; (**f**) influence of time and temperature on selectivity.

The most influential factor in yield is temperature, given its higher coded coefficient of 5.57, followed by time and FeCl_3_ concentration with similar coefficients of 2.15 and 1.98, respectively. As for the interactions between factors, it can be seen that the yield at a high temperature varies little with the concentration of FeCl_3_, being slightly higher at an intermediate concentration owing to the slight curvature it presents (quadratic term, A^2^), while at a low temperature, the yield increases with the concentration of FeCl_3_ (Figure 3a). In the interaction with time, the behaviour is similar to that already described; the performance decreases in a negligible way with the increase of time at a high temperature, while at a low temperature, it increases with time (Figure 3b). The yield is higher at a high temperature for both interactions. The conversion and the yield have similar behaviour in front of the studied factors, as can be seen in the obtained models (Table 4) and the response surfaces (Figure 3), sharing the temperature as the most influential factor. Analyzing more in detail, the conversion increases with the increase of FeCl_3_ concentration, as well as with the increase of time, with this increase being more accentuated at low temperatures in both cases (Figure 3c,d). At a high temperature, the highest conversion values occur, and it is at this point that the increase in time has little influence on the increase in conversion (Figure 3d).

Selectivity shows different behaviour than that found in yield and conversion (Figure 3). Selectivity has, as its most influential factor, the concentration of FeCl_3_, followed by time and temperature at a considerable distance, according to the values of their coefficients of 2.23, 1.19, and 1.07, respectively (Table 4). Furthermore, in the selectivity model, only the linear terms were significant and all of them have a negative sign, which indicates that the lowest conditions of the factors are going to be the most favourable ones.

Table 5 shows the results of the best conditions and the values obtained from the different responses (performance, conversion, and selectivity) for different optimization proposals, giving the same weight to the responses in the case of multiple optimizations. The highest value of selectivity would be 64.98%, and would be obtained under the lowest conditions of the design, that is, 190 °C, [FeCl_3_] = 0.05 M, and 0.5 min. On the contrary, the highest conversion value would be obtained under the highest design conditions, that is, 200 °C, [FeCl_3_] = 0.1 M, and 1 min, with a value of 95.93%. The highest yield value, 53.71%, is obtained at 200 °C, [FeCl_3_] = 0.07 M, and 0.5 min, a value close to that obtained under the conditions of maximizing conversion, 52.84%; on the contrary, the conversion and selectivity values are more distant.

Different optimizations have been made with more than one response, with yield as the common response in all cases. In the first of these, yield and selectivity have been jointly optimized, obtaining conditions of 200 °C, [FeCl_3_] = 0.05 M, and 0.5 min and values of 53.24% and 62.84%, respectively, these values being very close to the individual optimums. In the second one, performance and conversion have been jointly optimized, obtaining conditions of 200 °C, [FeCl_3_] = 0.1 M, and 1 min, resulting in these conditions having a greater difference compared with the individual performance optimum. Finally, in the case of the triple optimization of the three responses, the same conditions result as in the joint optimization of yield and selectivity, that is, 200 °C, [FeCl_3_] = 0.05 M, and 0.5 min, with a final yield of 53.24%. Reviewing the experimental results of the composite central design, it has been found that, under the conditions of one of the star points (202.07 °C, [FeCl_3_] = 0.075 M, and 0.75 min), the yield value obtained is on average 55.10% higher than that found under the optimum design conditions. The values of yield, conversion, and selectivity (54.81%, 92.62%, and 58.98%) according to the conditions of this star point were obtained with models resulting in errors of less than 1% (Table 5).

**Figure 4:**
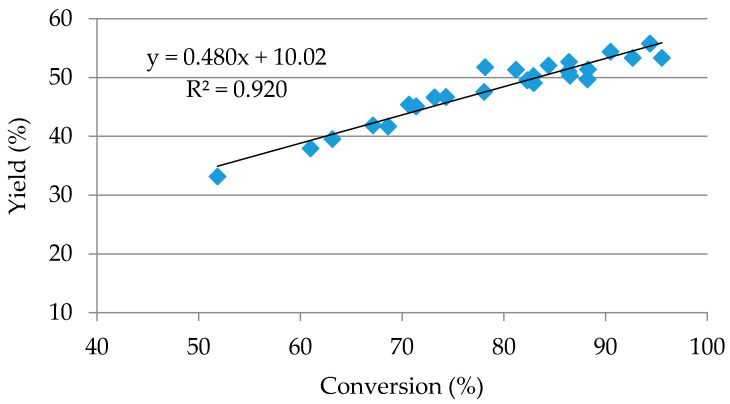
Linear performance regression versus conversion of the values of the composite central design.

As can be seen in Figure 4, the efficiency and conversion show a good linear adjustment with *R*^2^ = 0.92, resulting in a 58% efficiency for the 100% conversion, higher than the value obtained in the star point conditions. Given the above, using the model obtained for the conversion, the temperature for which 100% would be obtained was sought. The concentration of FeCl_3_ was set at 0.05 M and 0.5 min (triple optimum conditions) and a temperature of 210 °C was obtained (at 211 °C, the conversion is higher than 100%). The conditions of 210 °C, [FeCl_3_] = 0.05 M, and 0.5 min were tested experimentally, obtaining a conversion of 98.51%, very close to that predicted by the model of 99.34%. Under these conditions, a yield value of 57.12% was obtained, confirming the proposed hypothesis. This value is very close to 57.30%, which would result from substituting the conversion value of 98.51% in the regression of Figure 4. If the experimental values and those predicted by the models for this temperature condition outside the range are compared, the experimental values of yield and selectivity are lower, by 11.24% and 4.47%, respectively, and it can be said that the yield model cannot be used with temperatures so far from those in the initial range.

## 3. Discussion

Different authors have made designs of experiments to study the influence of different factors and to determine the best conditions to obtain the maximum performance of furfural. In the cases that are going to be considered next, the response surface methodology and quadratic models were used as in our work.

Lamminpää et al. [28] studied four variables by a central composite circumscribed design using a preheating oven (360–420 °C) and a fluidized sand bath. The variables were time (20–40 min), temperature (140–200 °C), initial xylose concentration (0.067–0.20 M), and formic acid concentration (10–30% *w*/*w*). The design contained 29 experiments, including five central points. In this design, the authors obtained conversions ranging from 6.2% to 98.2% and selectivities from 42% to 73%, values similar to those obtained in this work. Conversion and yield were mainly influenced positively by temperature and, as in our case, followed by acid concentration. The maximum yield value obtained was 65% at 200 °C, 20 min, 30 wt% of formic acid, and with an initial xylose concentration of 10 g/L. This yield in the same conditions, but increasing the initial xylose concentration to 30 g/L (the same as in our work), drops to 56.8%, very similar to the value obtained in our work of 57.1%, although with a much shorter time of only 0.5 min in our case. The industrial process adopted by China with corncobs achieves around 50% yield with 3–4 wt% sulfuric acid at 153 °C for 5 h [9].

Yang et al. [27] also performed a central composite design in this case in a stainless steel autoclave, with an electric jacket, a constant initial xylose concentration of 80 g/L, and varying the proportion of water-o-nitrotoluene. The reaction temperature (170–210 °C), formic acid concentration (5–25 g/L, pH_25 °C_ 2.32–1.96), o-nitrotoluene volume percentage (20–80 vol%), and residence time (40–200 min) were analyzed in the experimental design. The design consisted of 27 experiments, including three central points. The results included selectivities between 51.1 and 99.7% and yields between 20.3 and 71.2%, which are higher than those obtained in the previous design and those presented in this work. Among the factors studied in this design, temperature and o-nitrotoluene percentage are the most influential, with time and formic acid concentration having a low influence on yield and selectivity. Both yield and selectivity present a maximum of around 190 °C. On the contrary, the increase of o-nitrotoluene percentage (0 vol% to 80 vol%) was positive in both yield and selectivity, with increases from about 30% to 70% and 70% to 99%, respectively. The maximum furfural yield (74%) and selectivity (86%) were obtained in 75 min at 190 °C for 20 g/L formic acid concentration and 75 vol% o-nitrotoluene. This yield is higher than that obtained in our work, but the time is much higher, that is, 75 min compared with 0.5 min, and it also uses an organic solvent from petroleum, which makes the process less sustainable. To reduce the reaction time, the authors added different halides (NaCl, KCl, KBr, and KI) with a concentration of 0.5 M, achieving an optimum time in 60 min, or in other words, a reduction of 15 min. This time is still far from that obtained in our work, even though it achieved a slight improvement in performance and selectivity, for example, with NaCl, of 78% and 90%, respectively.

Another type of design was used to study the effect of lignin on the production of furfural from xylose (30 g/L), at D-optimal design [29]. In the design, four variables were included: three quantitative variables, time (20–80 min), temperature (160–180 °C), and initial lignin concentration (0–20 g/L); and one qualitative variable, the acid catalyst (10% *w*/*w*) formic acid or 0.2% *w*/*w* sulfuric acid (initial pH similar). The design contained 26 experiments, including 3 central points, plus 4 extra points, for a total of 30 experiments. The xylose conversion varied from 12% to 90% with the formic acid and from 6% to 85% with the sulfuric acid, values somewhat lower than those obtained in our work. The furfural yield varied from 9% to 58% in HCOOH and from 4% to 53% in H_2_SO_4_, in the order of our work. The selectivities were 65–80% and 63–76% in HCOOH and H_2_SO_4_, respectively, higher than those obtained in our work, with the least variation between large and small values compared with the other responses. The most influential factors in yield are temperature and time in both HCOOH and H_2_SO_4_ cases. The difference in the influence of lignin is found to be more significant in the case of H_2_SO_4_ than in HCOOH; for example, at 180 °C in sulfuric acid, the yield is higher than 50% without lignin, but in the presence of 20 g/L lignin, the yield is lower than 40%, whereas in formic acid, the yield stays about the same, at around 62%. The maximum yields are given at 180 °C, 80 min, and without lignin, with values around 64% and 56% with HCOOH and H_2_SO_4_, respectively, values in the order of those obtained in this work, but with a much longer time. The authors concluded that lignin affected furfural formation in two ways: (1) the lignin has an acid neutralization capacity (the maximum rise in pH was 0.13 units in formic acid and 0.23 units in sulfuric acid; time and temperature are insignificant parameters for the pH change response model), and (2) the lignin inhibits xylose dehydration into furfural (the neutralizing effect does not explain all of the decreases in conversion and yield, so some additional mechanism must be involved) [29]. Rasmussen et al. [30] concluded that there are at least three possible routes for furfural formation from xylose, and the reaction conditions determine which mechanism dominates.

### Overview of the Production of Furfural from Xylose

Table 6 shows a varied representation of the processes used to produce furfural from xylose (maximize furfural yield) in monophase systems with green solvents, as in our case, to have processes without petroleum derivatives.

Among those green solvents are water, seawater, gamma-valerolactone (GVL), GVL-water, dimethyl sulfoxide (DMSO), N,N-dimethylacetamide (DMA), and some ionic liquids ([emin]Br, [emim]HSO_4_, [bmim]PF_6_) that are not chloride based as they are considered toxic and corrosive [31]. In most cases, some kind of catalyst was used to increase the reaction speed and improve the yield in furfural.

These catalysts can be both homogeneous and heterogeneous. Among the homogeneous catalysts are inorganic acids (HCl, H_2_SO_4_); organic acids (formic acid, maleic acid); salts (FeCl_3_, SnCl_4_); heteropolyacid salts (MP34CsPW, H_3_PW_12_O_40_ (PW)); a combination of mineral acid + salt (HCl-NaCl, H_2_SO_4_-FeCl_3_), as in our case; or combinations of different salts (CrCl_2_-LiBr). Among the heterogeneous catalysts, we find zeolites (H-ZSM-5, H-Mordenite, H-Beta) or mesoporous acid-catalysts (MSHS-SO_3_H, Nafion 117). 

There is also a combination of homogeneous and heterogeneous catalysts, such as inorganic acid + zeolite (HCl-Sn-beta) or a polymer bound an acid + salt (PEG-OSO_3_H-MnCl_2_). As for the forms of heating used, they could be grouped into two large groups, conventional ones like autoclave, oil bath or oven preheating (360–420 °C), and a fluidized sand bath; and non-conventional ones, such as microwaves (our case) and supercritical systems.

The initial xylose concentration used (Table 6) shows a wide range from just 5 g/L to 200 g/L, with the average being around 50 g/L and the median of 30 g/L, which coincides with the value used in this work. The temperatures used range from 100 °C to 250 °C, with the average being around 170 °C and the median being 180 °C. If only microwave heating is taken into account, the temperature range is shorter, 170–210 °C, where the average is just over 193 °C and the median is 200 °C, showing that optimum temperatures with microwaves are higher when using other forms of heating. The times used also show a wide range from 0.5 min (this work) to 240 min, with an average of 70 min, while the average in the case of using microwaves around a third is about 25 min, showing itself as a faster process and one that would also save energy [17,34]. The yields obtained range from 14% to 87%, with the average being around 61%; meanwhile, taking into account only microwave heating, this average would be around 55%; the value obtained in this work of 57% is in the order of these averages. If all the values shown in Table 6 are analyzed to see how they affect the furfural yield, the following is found: the highest values are given to short times (less than 20 min), high temperatures (180–200 °C) (same trend found in this work), and the increase of the initial xylose concentration having a slight negative effect.

With the idea of being able to increase the yield of furfural, one of the proposals is the biphasic systems in such a way that furfural is produced in one phase and is selectively transferred to the insoluble organic phase, avoiding its degradation and displacing the reaction balance. Several authors have reported that they have doubled the performance of furfural when using a biphase system (with methyl isobutyl ketone (MIBK) or cyclopentylmethyl ether (CPME)) versus a monophase system with water only [10,17,49]. Table 7 shows some biphase processes with green solvents for the production of furfural. 

In these processes, mainly water, but also ionic liquids ([bmim]HSO_4_) were used as the reaction phase solvents and organic cosolvents as follows: methyl isobutyl ketone (MIBK), 2-methyltetrahydrofuran (2MTHF), cyclopentylmethyl ether (CPME), dimethyl sulfoxide (DMSO), 2-s-butilfenol (SBP), and tetrahydrofuran (THF). In most cases, some kind of catalyst was used, either a homogeneous catalyst such as inorganic acids (HCl), salts (SnCl_4_), a combination of mineral acid + salt (H_2_SO_4_-NaCl), combinations of different salts (FeCl_3_-NaCl, AlCl_3_-NaCl) or ionic liquids ([Sbmim]HSO_4_, [SbPy]BF_4_); or a combination of heterogeneous and homogeneous (Sn-MMT-NaCl, Nafion NR50-NaCl). As for the forms of heating used, the processes could be grouped into conventional ones, including autoclave and oil bath, and non-conventional ones such as microwaves. The initial xylose concentration assayed shows a very wide range from 18.5 g/L to more than 600 g/L, with the average being around 150 g/L, or around 120 g/L in the case of microwave heating. The range of temperature was from 130 °C to 180 °C, with the median being 170 °C, the same median value as in the case of microwave heating. Concerning the values of the process time, a wide range from 20 min to 360 min was used, with an average close to 110 min, with the average in the case of using microwaves around a third being about 39 min.

Microwave-assisted processes were faster than conventional heated processes, as in the case of monophasic, and also more selective [34]. The yields obtained ranged from 63% to 100%, with the average being around 80%, with the average value being very similar with microwave heating, at 79%. The average value obtained in biphasic processes is 30% higher than the average value obtained in monophasic processes (80% versus 60%). If all the values shown in Table 7 were analyzed to see how they affected the furfural yield, it is found that the highest values were given at high temperatures (170–180 °C) and at short times (less than 60 min), while the initial xylose concentration had no apparent influence.

## 4. Materials and Methods

### 4.1. Chemical Reagents

Xylose was purchased from Fagron (Terrassa, Spain); FeCl_3_ hexahydrate (99%) was purchased from Emsure (Darmstadt, Germany); H_2_SO_4_ (98%) was purchased from Honeywell Fluka (Seelze, Germany). All materials were used without further purification. Aqueous solutions were prepared with deionized H_2_O.

### 4.2. General Procedure for Dehydration Treatment

All experiments were conducted using a microwave reactor (Anton Paar Monowave 400, Graz, Austria). The xylose and the catalysts in different concentrations were charged in the 10 mL glass vessel. The reaction volume was 4 mL; the xylose concentration was 30 g/L; and the catalysts used were H_2_SO_4_ at 2% *w*/*v* acting as Brønsted acid and FeCl_3_ as Lewis acid, with a variable concentration [10,13].

The heating dynamic followed was to heat the sample to the set temperature in 2 min, maintaining the temperature for the experiment time, and cooling down to 40 °C with compressed air. The magnetic agitation during the heating and maintenance was 600 rpm, and it was 800 rpm during the cool down period. The temperature was measured by an IR sensor (Anton Paar Monowave 400, Graz, Austria). The pressure inside the glass vessel was also monitored throughout the experiments, through the septum that covers it. The experimental conditions were set based on previous experiments not included.

### 4.3. Methodology Based on the Design of Experiments

A two-level factorial design with three central points was carried out. The FeCl_3_ concentration in the range of 0.1 and 0.3 M, the process time from 1 to 5 min, and the temperature between 170 and 200 °C were selected as factors, with a total of 11 experiments (Table 1).

The samples obtained in the microwave reactor were analyzed in high performance liquid chromatography (HPLC), as detailed in Section 4.4, and the results in terms of xylose conversion, furfural yield, and selectivity, as defined below, were taken as responses and statistically analyzed with the commercial software Design Expert 7.0.0, Stat-Ease Inc (Minneapolis, MN, USA).

On the basis of the data obtained from this first factorial design, a second rotatable composite central design was performed, where the range of variation for the factors was reduced as follows: the concentration of FeCl_3_, 0.05–0.1 M; time, 0.5–1 min; and temperature, 190–200 °C. Moreover, five center and star points (values above and below the experimental range for each of the factors) were added (Table 3).

The liquors obtained were measured in HPLC and analyzed statistically as in the previous design. These results were optimized to obtain the maximum yield of furfural and selectivity.

### 4.4. Analysis of the Liquid Fractions and Quantification of the Yield, Selectivity, and Conversion

The liquors obtained in the microwave reactor were analyzed by high performance liquid chromatography (HPLC). The compounds were determined using an Agilent Technologies 1260 model (Santa Clara, CA, USA) with ICSep ICE-COREGEL 87H3 column operating at 65 °C with 5 mM sulfuric acid as the mobile phase (0.6 mL/min). Samples were previously filtered through 0.45 μm nylon membranes.

Conversion of xylose, furfural selectivity, and yield were defined as follows:

(1) $$\mathrm{Xylose}\;\mathrm{conversion}\;\left(\%\right)=\frac{\mathrm{Consumed}\;\mathrm{xylose}}{\mathrm{Initial}\;\mathrm{xylose}}\times 100$$

(2) $$\mathrm{Furfural}\;\mathrm{yield}\;\left(\%\right)=\frac{\mathrm{Furfural}\;\mathrm{produced}}{\mathrm{Furfural}\;\mathrm{stoichiometric}\;\mathrm{potential}\left(*\right)}\times 100$$

(*) Furfural stoichiometric potential = Initial xylose × 0.64(3)

(4) $$\mathrm{Xylose}\;\mathrm{conversion}\;\left(\%\right)=\frac{\mathrm{Consumed}\;\mathrm{xylose}}{\mathrm{Initial}\;\mathrm{xylose}}\times 100$$

## 5. Conclusions

The analysis and comparison of results demonstrated the following:

Furfural can be obtained by dehydration of xylose, the main pentose found in lignocellulosic materials, thus making use of these renewable sources of energy and biobased chemicals. Temperature, time, and the concentration of iron chloride were identified as relevant factors of the conversion process. Following a two experimental design approach and optimization by response surface methodology, a highest yield of 57.1% along with 98.5% conversion was obtained. The experimental conditions leading to the best result, including the highest temperature assayed (210 °C) and lowest iron chloride concentration (0.05 M) and process time (0.5 min), can be used as a starting point for the study of the conversion process using the whole lignocellulosic material, after being subjected to the required operations.

The processes used to obtain xylose-rich liquors from lignocellulosic materials should be selective on the hemicelluloses to solubilize the minimum amount of lignin, as this negatively affects the production of furfural.

The use of microwave heating in both monophase and biphase systems allows shorter reaction times, around one-third compared with conventional heating methods, which saves time and probably also energy.

The highest furfural yield values are obtained at high temperatures and at short times in both monophase and biphase systems.

Biphase systems increase the furfural yield on average by about 30% compared with monophase systems, in each case, using green solvents.

Future investigations will be focused on the use of microwaves for heating and a biphase system with green solvents on a xylose-rich liquor obtained from lignocellulosic materials. The methodology found here to obtain the optimal conditions of temperature, time, and concentration of catalyst will be applied to obtain furfural with a high yield. According to the current results, we would propose that high temperatures, above 200 °C, should be tested.

## Figures and Tables

**Table 1:** Two-level, three-factor experimental design. Experimental conditions and response results.

Run	Factors			Responses				
	A: FeCl_3_ (M)	B: Time (min)	C: Temp. (°C)	Xylose Consumed (g/L)	Furfural (g/L)	Yield(%)	Conversion (%)	Selectivity (%)
2	0.3	1	170	16.68	4.79	24.87	55.61	44.73
4	0.1	5	200	29.33	7.84	38.99	97.77	39.88
6	0.1	5	170	18.11	6.52	33.87	60.38	56.10
7	0.3	1	200	29.44	7.96	41.13	98.13	41.91
8	0.3	5	170	26.75	7.72	39.93	89.15	44.79
9	0.1	1	200	29.07	10.35	53.59	96.91	55.30
10	0.1	1	170	7.52	3.02	15.63	25.06	62.38
11	0.3	5	200	28.99	4.82	24.96	96.63	25.83
1	0.2	3	185	29.04	8.91	45.61	96.80	47.12
3	0.2	3	185	29.13	9.05	45.70	97.12	47.06
5	0.2	3	185	28.55	9.16	45.71	95.18	48.02

**Table 2:** Models in coded terms obtained for yield, conversion, and selectivity in a factorial design. A = concentration of FeCl_3_, B = time, and C = temperature. CV, coefficient of variation.

Response	Model (Coded Terms)	*p*-Value	*R* ^2^	CV (%)	SD (%)
Yield (%)	Yield = 34.12 − 1.40·A + 0.32·B + 5.55·C − 5.22·AC − 8.01·BC	<0.0001	0.997	2.38	0.89
Conversion (%)	Conversion = 77.45 + 7.43·A + 8.53·B + 19.91·C − 7.41·AC − 8.69·BC	<0.0001	0.999	1.26	1.04
Selectivity (%)	Selectivity = 46.36 − 7.05·A − 4.71·B − 5.63·C − 3.16·BC	<0.0001	0.988	3.18	1.48

**Table 3:** Results (consumed xylose, furfural produced, yield, conversion, and selectivity) of the rotatable composite central design (duplicates of the star points +5 central points).

Run	A: FeCl_3_ (M)	B: Time (min)	C: Temp. (°C)	Consumed Xylose (g/L)	Furfural (g/L)	Yield (%)	Conversion (%)	Selectivity (%)
1	0.040	0.75	195	21.24	8.73	45.39	70.68	64.22
2	0.1	1	190	23.60	10.01	51.77	78.16	66.23
4	0.05	0.5	200	25.43	10.04	52.07	84.40	61.69
5	0.05	1	190	20.31	8.11	41.87	67.14	62.36
6	0.1	1	200	28.88	10.32	53.35	95.54	55.84
7	0.075	1.10	195	26.66	9.93	51.38	88.28	58.20
8	0.075	0.75	202.07	28.45	10.76	55.78	94.37	59.11
9	0.075	0.75	187.93	19.07	7.64	39.55	63.15	62.63
11	0.1	0.5	200	27.97	10.30	53.35	92.68	57.56
12	0.040	0.75	195	22.05	8.99	46.64	73.18	63.73
14	0.110	0.75	195	26.64	9.61	49.69	88.22	56.33
15	0.075	0.40	195	21.56	8.72	45.10	71.38	63.19
17	0.05	0.5	190	15.57	6.37	33.16	51.85	63.96
18	0.110	0.75	195	25.98	9.67	50.30	86.51	58.15
19	0.075	0.75	187.93	18.32	7.29	37.95	61.00	62.22
20	0.075	0.40	195	22.31	8.97	46.68	74.31	62.82
21	0.1	0.5	190	20.73	8.06	41.71	68.63	60.78
22	0.05	1	200	26.09	10.18	52.67	86.40	60.95
23	0.075	0.75	202.07	27.28	10.50	54.41	90.50	60.12
25	0.075	1.10	195	26.06	9.86	51.05	86.35	59.12
3	0.075	0.75	195	25.08	9.73	50.27	82.91	60.63
10	0.075	0.75	195	25.05	9.49	49.08	82.94	59.18
13	0.075	0.75	195	24.73	9.53	49.54	82.30	60.19
16	0.075	0.75	195	24.50	9.91	51.33	81.20	63.22
24	0.075	0.75	195	23.54	9.18	47.56	78.06	60.93

**Table 4:** Models in coded and real terms obtained for performance, conversion, and selectivity in the rotatable composite central design. A = concentration of FeCl_3_, B = time, and C = temperature.

Response	Model (Coded and Real Terms)	*p*-Value	*R* ^2^	CV (%)	SD (%)
Yield (%)	(Coded) = 49.10 + 1.98·A + 2.15·B + 5.57·C − 2.06·AC − 2.27·BC − 0.54·A^2^ − 1.09·C^2^ (Real) = −2345.52 + 3423.72·A + 362.70·B + 20.67·C − 16.48·AC − 1.82·BC − 871.20·A^2^ − 0.04·C^2^	<0.0001	0.970	2.38	1.14
Conversion (%)	(Coded) = 80.34 + 5.55·A + 4.41·B + 11.20·C − 1.30·AC − 2.50·BC − 1.78·C^2^ (Real) = 3533.74 + 2246.99·A + 406.90·B + 32.25·C − 10.38·AC − 2.00·BC − 0.07·C^2^	<0.0001	0.977	2.46	1.95
Selectivity (%)	(Coded) = 60.49 − 2.23·A − 1.19·B − 1.07·C (Real) = 112.51 − 89.22·A − 4.74·B − 0.21·C	<0.0001	0.911	1.26	0.77

**Table 5:** Results of the optimization of the rotatable composite central design.

Maximise	A: FeCl_3_ (M)	B: Time (min)	C: Temp. (°C)	Yield (%)	Conversion (%)	Selectivity (%)
Yield	0.07	0.5	200	53.71	87.53	60.77
Conversion	0.1	1	200	52.84	95.93	56.01
Selectivity	0.05	0.5	190	33.44	53.61	64.98
Yield and selectivity	0.05	0.5	200	53.24	83.59	62.84
Yield and conversion	0.1	1	200	52.84	95.93	56.01
Yield, conversion, and selectivity	0.05	0.5	200	53.24	83.59	62.84
Star point experimental average	0.075	0.75	202.07	55.10	92.43	59.62
Predict star point	0.075	0.75	202.07	54.81	92.62	58.98
Out of range	0.05	0.5	210	64.35	99.34	60.70
Experimental out of range	0.05	0.5	210	57.12	98.51	57.98

**Table 6:** Yield results of furfural production from xylose in green solvents in the monophase system.

Xylose	Solvent	Catalyst	Temperature/Time	Heating	Furfural Yield (%)	Ref.
30 g/L= 200 mM	H_2_O	MSHS-SO_3_H (3.3 g/L)	190 °C/1 h	Autoclave	43.5	[32]
10 wt%	H_2_O	H-ZSM-5 (catalyst-xylose ratio, 0.3 *w*/*w*)	200 °C/20 min	Autoclave	46	[33]
35 mM	H_2_O	HCl (50 mM)-NaCl (850 mM)	200 °C/5 min	Oil bath	81.3	[14]
35 mM	H_2_O	HCl (50 mM)-NaCl (3.5 wt% = 599 mM)	200 °C/440 s (7.3 min)	Microwave	76	[34]
740 mM	H_2_O	HCl (100 mM)	170 °C/30 min	Microwave	40	[17]
667 mM	H_2_O	HCl (100 mM)	180 °C/30 min	Microwave	39	[19]
67 mM = 10 g/L	H_2_O	Maleic acid (250 mM)	200 °C/28 min	Microwave	67	[35]
57 mM	H_2_O	Not used	200 °C/60 min	Microwave	49	[36]
30 g/L	H_2_O	H_2_SO_4_ (2% *w*/*v*)-FeCl_3_ (0.05 M)	210 °C/0.5 min	Microwave	57.1	Present study
67 mM	H_2_O	Formic acid (30 wt%)	200 °C/20 min	Oven preheating (360–420 °C) and a fluidized sand bath	65	[28]
200 mM	H_2_O	Formic acid (30 wt%)	200 °C/20 min	Oven preheating (360–420 °C) and a fluidized sand bath	56.8	[28]
30 g/L	H_2_O	Formic acid (30 wt%)	180 °C/80 min	Oven preheating (360–420 °C) and a fluidized sand bath	~63.8	[29]
30 g/L	H_2_O	H_2_SO_4_ (0.2 wt%)	180 °C/80 min	Oven preheating (360–420 °C) and a fluidized sand bath	~56.2	[29]
18 wt%	H_2_O	H_2_SO_4_ (20 mM)	250 °C/1 min	Supercritical flow reactor system	64	[37]
50 mM	Seawater (salts (26,46 g/kg)	HCl (50 mM)	200 °C/10 min	Oil bath	71.7	[38]
150 mM	GVL	H_2_SO_4_	175 °C	Not specified	75	[39]
2.4 wt%	GVL	FeCl_3_·6H_2_O (0.6 wt%)	180 °C/9 min	Oil bath	83.6	[13]
2 wt%	GVL-H_2_O (10 wt% H_2_O)	H_2_SO_4_ (0.05 M)	170 °C/15 min	Oil bath	87	[40]
2 wt%	GVL-H_2_O (10 wt% H_2_O)	H-Mordenite	175 °C/2 h	Oil bath	81	[41]
2 wt%	GVL-H_2_O (10 wt% H_2_O)	H-Beta (3.75 wt%)	160 °C/1 h	Oil bath	71	[40]
200 mM	DMSO	H-Mordenite (100 g/L)	140 °C/4 h	Autoclave	39	[42]
200 mM	DMSO	MP34CsPW (30 g/L)	140 °C/4 h	Oil bath	45	[3]
200 mM	DMSO	H_3_PW_12_O_40_ (PW) (20 g/L)	140°C/4 h	Oil bath	67	[43]
9.1 wt%	DMSO	Nafion 117 (20 wt% of initial xylose)	150 °C/2 h	Oil bath	60	[44]
10 wt%	DMSO	HCl (0.1 M)-Sn-beta	110 °C/3 h	Not specified	14.3	[45]
10 wt%	DMA	CrCl_2_ (6 mol% of xylose)-LiBr (10 wt%)	100 °C/4 h	Oil bath	56	[46]
20 wt%	[emin]Br	SnCl_4_ (10 mol% of xylose)	130 °C/1 h	Oil bath	71.1	[15]
100 g/L	[emim]HSO_4_	Not used	100 °C/30 min	Oil bath	62	[47]
37.5 g/L	[bmim]PF_6_	PEG-OSO_3_H (50 mM)-MnCl_2_ (75 mM)	120 °C/18 min	Not specified	75	[48]

GVL, gamma-valerolactone; DMSO, dimethyl sulfoxide; DMA, N,N-dimethylacetamide; PEG, polyethylene glycol.

**Table 7:** Yield results of furfural production from xylose in green solvents in biphase systems.

Xylose	Solvent	Catalyst	Temperature/Time	Heating	Furfural Yield (%)	Ref.
1.85 wt%	[bmim]HSO_4_-MIBK (1:4.4, *w*/*w*)	Not used	140 °C/4 h	Oil bath	80.3	[50]
400 mmol/L H_2_O	H_2_O-2MTHF (1:1, *v*/*v*)	FeCl_3_ (80 mM)-NaCl (20 wt%*)*	140 °C/4 h	Oil bath	71	[51]
4 wt% H_2_O phase	H_2_O-CPME (1:2.33, *v*/*v*)	H_2_SO_4_ (1 wt% H_2_O phase)-NaCl (40 wt% H_2_O phase)	170 °C/100 min	Oil bath	100	[52]
1.25 mol/L H_2_O	H_2_O-CPME (1:3, *v*/*v*)	FeCl_3_ (5.08 g/L)-NaCl (18.13 g/L)	170 °C/20 min	Microwave	74	[10]
1 mol/L H_2_O	H_2_O-CPME (1:3, *v*/*v*)	NaCl (23.75 g/L)-Nafion NR50 (23.75 g/L)	170 °C/40 min	Microwave	80	[49]
200 g/L H_2_O	H_2_O-DMSO (1:1, *v*/*v*)	SnCl_4_ (catalyst/xylose molar ratio 0.5)	130 °C/6 h	Oil bath	63	[53]
10 wt% H_2_O phase	H_2_O-DMSO-SBP (5:1:5, *v*/*v*/*v*)	Sn-MMT (xylose-catalyst, 5:1 *w*/*w*)-NaCl (satured solution, aprox. 36 g/100 g of H_2_O)	180 °C/30 min	Autoclave	76.8	[54]
1 g/L,5 mL H_2_O	H_2_O-MIBK (1.5:8, *v*/*v*)	[Sbmim]HSO_4_ (0.5g/1.5 mL H_2_O)	150 °C/25 min	Autoclave	91.4	[55]
740 mmol/L H_2_O	H_2_O-MIBK (1:1, *w*/*w*)	HCl (0.1 mol/L H_2_O)	170 °C/30 min	Microwave	80	[17]
10 wt% of H_2_O	H_2_O-THF (1:2, *w*/*w*)	[SbPy]BF_4_ (100 wt% of initial xylose)	180 °C/1 h	Microwave	85	[56]
250 mmol/L H_2_O	H_2_O-THF (1:3, *v*/*v*)	AlCl_3_-6H_2_O (25 mM)-NaCl (1.5 M)	140 °C/45 min	Microwave	75	[57]

MIBK, methyl isobutyl ketone; 2MTHF, 2 methyltetrahydrofuran; CPME, cyclopentylmethyl ether; DMSO, dimethyl sulfoxide; SBP, 2-s-butilfenol; THF, tetrahydrofuran.
